# Zebrafish mutants provide insights into Apolipoprotein B functions during embryonic development and pathological conditions

**DOI:** 10.1172/jci.insight.130399

**Published:** 2021-07-08

**Authors:** Hanoch Templehof, Noga Moshe, Inbal Avraham-Davidi, Karina Yaniv

**Affiliations:** Department of Biological Regulation, Weizmann Institute of Science, Rehovot, Israel.

**Keywords:** Development, Vascular Biology, Endothelial cells, Lipoproteins

## Abstract

Apolipoprotein B (ApoB) is the primary protein of chylomicrons, VLDLs, and LDLs and is essential for their production. Defects in ApoB synthesis and secretion result in several human diseases, including abetalipoproteinemia and familial hypobetalipoproteinemia (FHBL1). In addition, ApoB-related dyslipidemia is linked to nonalcoholic fatty liver disease (NAFLD), a silent pandemic affecting billions globally. Due to the crucial role of APOB in supplying nutrients to the developing embryo, *ApoB* deletion in mammals is embryonic lethal. Thus, a clear understanding of the roles of this protein during development is lacking. Here, we established zebrafish mutants for 2 *apoB* genes: *apoBa* and *apoBb.1*. Double-mutant embryos displayed hepatic steatosis, a common hallmark of FHBL1 and NAFLD, as well as abnormal liver laterality, decreased numbers of goblet cells in the gut, and impaired angiogenesis. We further used these mutants to identify the domains within ApoB responsible for its functions. By assessing the ability of different truncated forms of human APOB to rescue the mutant phenotypes, we demonstrate the benefits of this model for prospective therapeutic screens. Overall, these zebrafish models uncover what are likely previously undescribed functions of ApoB in organ development and morphogenesis and shed light on the mechanisms underlying hypolipidemia-related diseases.

## Graphical abstract

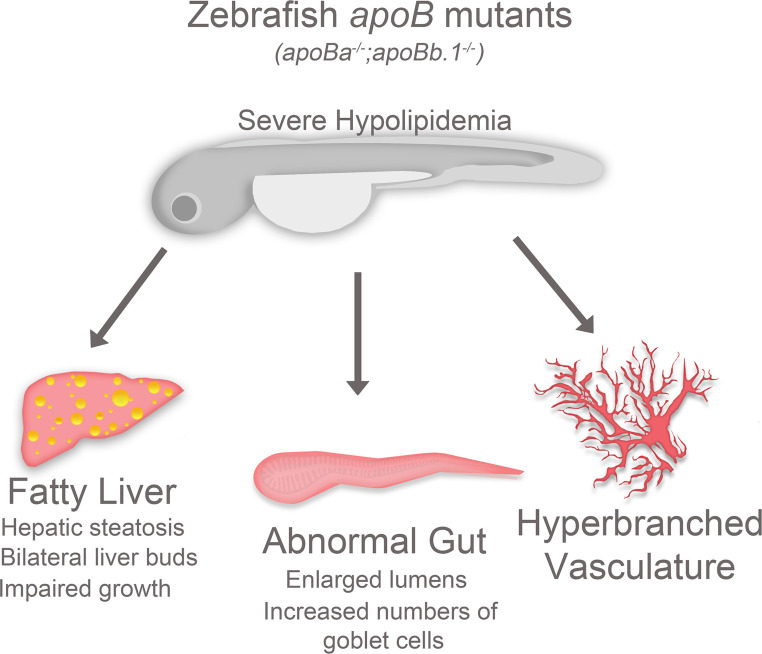

## Introduction

Apolipoprotein B (ApoB) is the primary structural component of atherogenic lipoproteins, such as chylomicrons, VLDLs, and LDLs, and is essential for their assembly (1). Elevated levels of LDL are widely recognized as a cardiovascular risk factor, and abundant data point to chemically modified LDL and ApoB as triggers of most of the features of the pathobiology of atherosclerosis. Besides the important role of APOB in lipid metabolism, a large body of data, accumulated during the past years, has revealed new roles for lipoproteins as signaling mediators in various cell types, operating at different levels and through various classic and nonclassic mechanisms (2).

Two APOB isoforms, APOB100 and APOB48, are present in humans and mice, which are encoded by a single gene and generated by RNA editing (3). Defects in ApoB synthesis and secretion, resulting from mutations in the *ApoB*, microsomal triglyceride transfer protein (*MTP*), and proprotein convertase subtilisin/kexin type 9 genes, lead to abetalipoproteinemia (ABL) and familial hypobetalipoproteinemia (FHBL; refs. 4, 5), two disorders characterized by low or absent levels of ApoB and LDL-cholesterol in plasma. ABL and homozygous FHBL display similar clinical symptoms, including steatorrhea, neurological dysfunction, ophthalmologic abnormalities, and fatty liver. Since both disorders also carry a reduced risk of cardiovascular diseases (CVDs), research into the underlying molecular mechanisms could uncover attractive targets for lipid-lowering therapies in patients with hypercholesterolemia.

In addition to the involvement of ApoB in atherosclerosis and CVD, ApoB-related dyslipidemia is linked to another global epidemic, nonalcoholic fatty liver disease (NAFLD), which affects around 20%–30% of the adult population in Western countries (6). NAFLD results in steatosis, the accumulation of lipid in hepatocytes (7), which can lead to liver malfunction, cirrhosis, and, in some cases, hepatocellular carcinoma (8). Defects in production and/or secretion of ApoB lipoproteins are highly linked to hepatic steatosis (9). In particular, the impaired synthesis of VLDL observed in patients with FHBL, which results in triglyceride accumulation in the liver, represents one among many causes of NAFLD in humans (10). The crucial role ApoB plays in various pathological conditions has prompted the generation of several genetically modified mouse models (11, 12). However, because APOB is expressed in the yolk sac during early embryonic development, where it facilitates the supply of nutrients to the developing embryo, ApoB-knockout mice die at gestational stage 9.5 to 10.5, thereby precluding the study of the roles of this protein during embryogenesis.

The close relationship between zebrafish and human apolipoprotein expression and function (13, 14) makes the former an attractive animal model for studying the repertoire of ApoB functions during embryonic development. In addition, the well-documented ability of zebrafish to develop hyperlipidemia (15, 16) and lipoprotein oxidation (17, 18) motivated the study of the cellular and molecular mechanisms linking lipoproteins and CVD in this animal. Here, we established mutants for 2 zebrafish *apoB* genes: *apoBa* and *apoBb.1*. We found that double-mutant embryos displayed lipid accumulation in hepatocytes, a common hallmark of FHBL1 and NAFLD. In addition, we detected early developmental defects, including abnormal liver laterality, decreased numbers of goblet cells in the gut, and impaired angiogenesis in double *apoB* mutants. Interestingly, we found altered Notch signaling to underlie some of the observed phenotypes. To identify the domains within ApoB associated with the antiangiogenic function, we assessed the ability of truncated forms of the human APOB (APOB25 and APOB34) to rescue the zebrafish mutant phenotypes. We found that only mutant embryos injected with APOB34 displayed a significant rescue of the vascular phenotypes, suggesting that the antiangiogenic activity of ApoB is contained between the 25 to 34 N-terminus remnant of the ApoB protein. Altogether, these results uncover previously unappreciated functions of ApoB during early organ development and highlight the potential of these newly established *apoB* zebrafish mutants as models for studying human pathologies associated with hypolipidemia, as well as for related drug screens.

## Results

### Generation of zebrafish apoB mutants.

**Figure 1:**
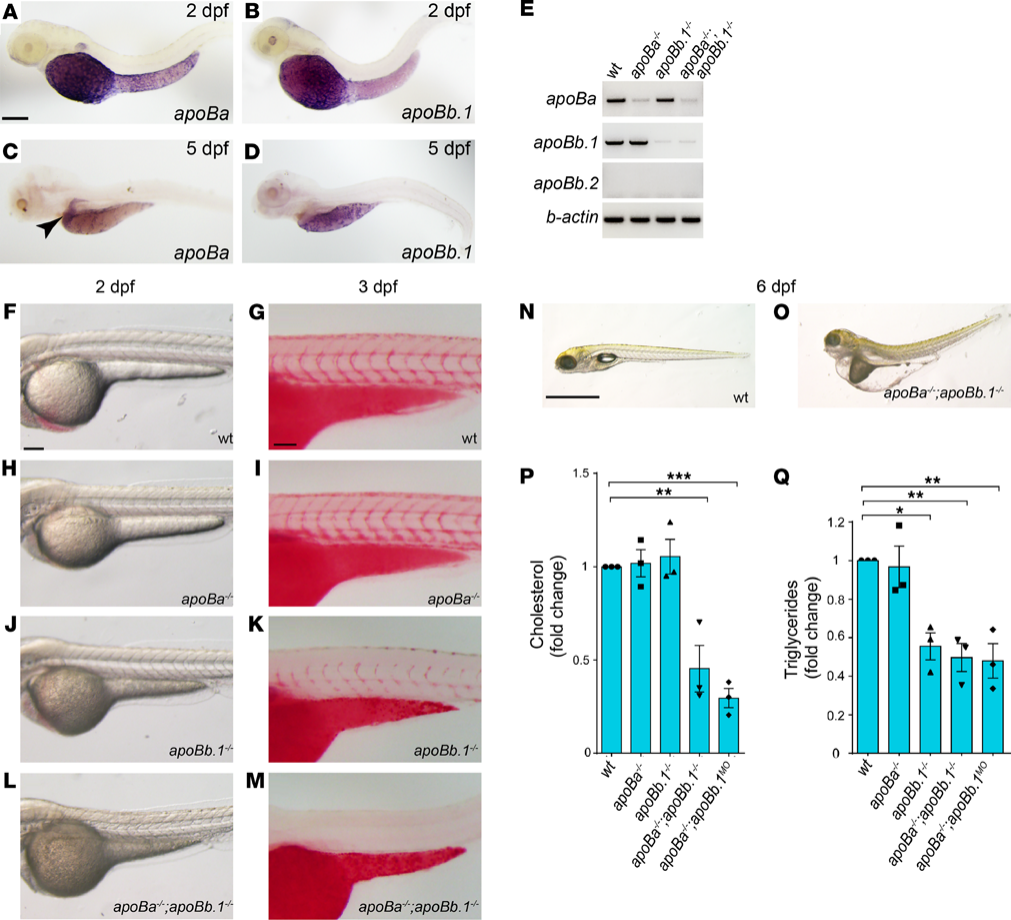
*apoB* mutants feature severe hypolipidemia. (**A**–**D**) Whole mount in situ hybridization (WISH) on 2 dpf zebrafish embryos showing strong expression of *apoBa* (**A**) and *apoBb.1* (**B**) in the YSL (*n_apoBa_* = 7; *n_apoBb.1_* = 6). At 5 dpf *apoBa* expression is enriched in the liver (**C**) and *apoBb.1* in the YSL and the intestine (**D**) (*n_apoBa_*=3; *n_apoBb.1_* = 3). (**E**) Semiquantitative PCR for the *apoB* genes in the different mutants. (**F**–**M**) Transmitted light images of 2 dpf embryos showing dark yolk in *apoBb.1^–/–^* (**J**) and *apoBa^–/–^ apoBb.1^–/–^* (**L**) but not in WT (**F**) or *apoBa^–/–^* (**H**) mutant embryos. Three dpf embryos stained with Oil Red O (ORO) show decreased lipid levels in *apoBb.1* mutant (**K**), as compared with WT (**G**) and *apoBa^–/–^* (**I**), embryos. (**M**) *apoBa apoBb.1* double mutants display complete absence of lipids in circulation. (**N** and **O**) Transmitted light images at 6 dpf demonstrate severe malformations, unabsorbed yolk, and pronounced edema in *apoBa apoBb.1* double mutants as compared with WT siblings. (**P** and **Q**) Cholesterol (**P**) and TG (**Q**) levels in the different *apoB* mutants compared with WT controls at 3 dpf. All measurements were carried out in deyolked embryos. *n* *=* 3, *n*_embryos/sample_ = 20. The data are shown as the mean ± SEM, calculated using ANOVA followed by Tukey’s multiple-comparison test. Scale bar: (**A**–**D**; **F**–**M**) 100 μm, (**N** and **O**) 1 mm. *P < 0.05, **P < 0.01, ***P < 0.001. *P* *<* 0.05 (considered significant versus control group).

Three *apoB* genes are detected in the zebrafish genome: *apoBa*, *apoBb.1*, and *apoB.2* (14, 19). Two days postfertilization (dpf) embryos displayed strong expression of *apoBa* and *apoBb.1* mRNA in the yolk syncytial layer (YSL), whereas *apoBb.2* expression was undetectable (Figure 1, A, B, and E). At 5 dpf, *apoBa* expression became restricted to the liver (Figure 1C, arrowhead), and *apoB.1* was detected mostly in the YSL and the intestine (Figure 1D). To investigate the role of ApoB during embryonic development, we generated zebrafish carrying mutations in the *apoBa* and *apoBb.1* genes. sgRNAs targeting exon 5 of the *apoBb.1* gene (Supplemental Figure 1A; supplemental material available online with this article; https://doi.org/10.1172/jci.insight.130399DS1) were injected along with *cas9* mRNA into 1-cell-stage *Tg(fli1:EGFP)* embryos (20). F0 injected fish were raised to adulthood and screened for germline transmission. We identified 2 different mutations, including an 18 bp insertion containing a premature in-frame stop-codon (Supplemental Figure 1A), which was used throughout this study. To target the *apoBa* gene, we used the transcription activator-like effector nuclease (TALEN) technology. mRNA-encoding left and right TALENs designed to hit exon 3 of the *apoBa* gene (Supplemental Figure 1B) were injected into 1-cell-stage *Tg(fli1:EGFP)* embryos. We identified 3 mutations, out of which we chose to focus on an 8 bp deletion in the TALENs’ target site, introducing a premature stop-codon after 70 amino acids (Supplemental Figure 1B). Both *apoBa* and *apoBb.1* mutants displayed strong decreases of the respective mRNA levels (Figure 1E). In addition, Western blot analysis demonstrated complete absence of ApoB in *apoBb.1* but not in *apoBa* mutants (Supplemental Figure 1C), confirming previous data showing that ApoBb.1 is the predominant isoform (~95% abundance; ref. 14).

### Characterization of the mutant phenotypes.

*apoBa* homozygous mutants were viable, fertile, and morphologically indistinguishable from their WT siblings (Figure 1, F and H, and Supplemental Figure 2A). Moreover, lipid distribution was normal in these mutants, as shown by Oil Red O (ORO) staining (ref. 15 and Figure 1, G and I). Similarly, injection of *apoBa* antisense morpholino oligonucleotides (MOs) did not elicit any noticeable phenotypes (Supplemental Figure 2, D and E). In contrast, *apoBb.1* homozygous mutants presented with several defects. First, they were easily identified due to their dark yolk (Figure 1J and Supplemental Figure 2B), indicative of impaired lipid absorption and aberrant accumulation of lipid droplets in the YSL (15, 21). This was also verified by ORO staining, which showed a strong reduction in circulating lipid levels (Figure 1K). Injection of *apoBb.1* MOs into WT embryos fully phenocopied the mutant phenotype (Supplemental Figure 2F). However, no effects were observed following injection of *apoBb.1* MOs into *apoBb.1* mutants (Supplemental Figure 2, G and H), confirming the MO specificity. The central role of the *apoBb.1* isoform was further corroborated by the decreased viability of the *apoBb.1* mutants (Supplemental Figure 2L). In addition, we noticed that the few *apoBb.1^–/–^* animals that survived by approximately 60 dpf were significantly thinner than their WT counterparts (Supplemental Figure 2M).

Although ApoBb.1 represents the predominant ApoB isoform, we suspected that the presence of ApoBa in *apoBb.1* mutants could compensate for the lack of its functions. Therefore, we generated *apoBa* *apoBb.1* double homozygous mutants (Figure 1L and Supplemental Figure 2C). These animals displayed a darker yolk and complete absence of lipids in circulation (Figure 1M). Moreover, the lack of both ApoB isoforms led to lethality at 6 to 8 dpf, with the larvae exhibiting profound yolk and pericardial edema, as well as a curved trunk (Figure 1, N and O). Finally, injection of *apoBb.1* MOs into *apoBa* homozygous mutants or vice versa (*apoBa* MOs into *apoBb.1^–/–^* embryos) recapitulated the phenotypes of *apoB* double mutants (Supplemental Figure 1C and Supplemental Figure 2, I–K).

We then carried out a lipid profile analysis of the different mutants. In order to accurately measure yolk-to-body ApoB-dependent transport, we utilized deyolked embryos. As seen in Figure 1P, we found no differences in cholesterol levels between *apoBa^–/–^* and *apoBb.1^–/–^* individual mutants. In contrast, these were strongly reduced in both *apoBa apoBb.1* double mutants and *apoBa^–/–^ apoBb.1^MO^*, as compared with WT siblings (Figure 1P). Interestingly, triglyceride (TG) measurements revealed a slightly different picture (Figure 1Q). While in *apoBa^–/–^* mutants TG levels were similar to those of WT siblings, levels were strongly decreased in *apoBb.1* mutants (Figure 1Q). In addition, TG levels were significantly reduced in both *apoBa apoBb.1* double mutants and in *apoBa^–/–^ apoBb.1^MO^* (Figure 1Q).

Due to the difficulty in obtaining large numbers of double homozygous mutants from *apoBa^–/–^* × *apoBb.1^+/–^* crosses, and having verified that the *apoBb.1* MO fully recapitulates the mutant phenotype, we decided to use *apoBa^–/–^ apoBb.1^MO^* embryos in most experiments of this study.

### Defective organ development in apoB mutants.

**Figure 2:**
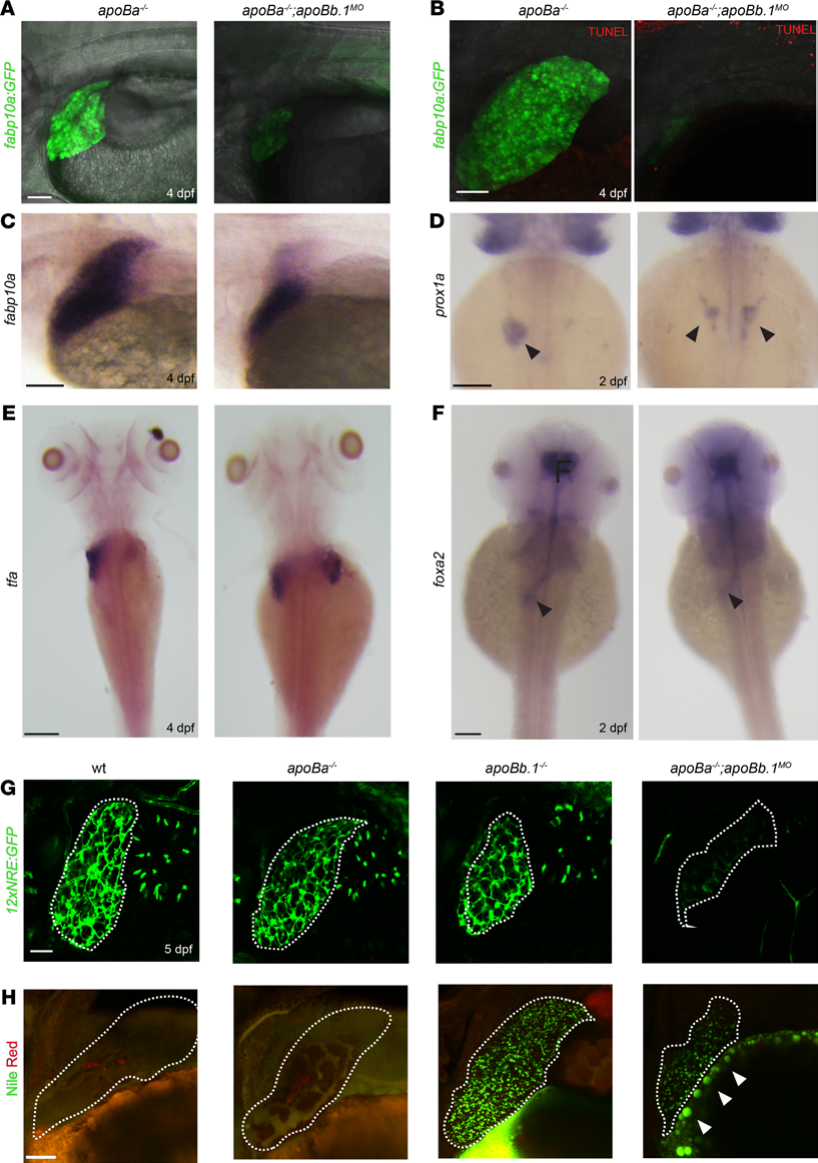
*apoB* mutants exhibit defective liver development and steatosis. (**A**) Confocal images at 4 dpf of *apoBa*^–/–^ and *apoBa*^–/–^ *apoBb.1^MO^* embryos in *Tg(-2.8fabp10a:EGFP)^as3^* background, showing smaller liver and decreased expression of *fabp10a EGFP* in *apoBa*^–/–^ *apoBb.1^MO^* embryos (*n*_WT_ = 8/8, *n_apob–/– apoBa–/– apoBb.1MO_* = 7/7). (**B**) No apoptotic cells are detected in the liver of *apoBa*^–/–^ or *apoBa*^–/–^ *apoBb.1^MO^* embryos, following TUNEL staining (*n*_WT_ = 6/6, *n_apoBa–/– apoBb.1MO_* = 5/5). (**C**) WISH showing expression of *fabp10a* at 4 dpf, depicting decreased liver size in *apoBa*^–/–^ *apoBb.1^MO^* embryos (*n*_WT_ = 7, *n_apoBa–/– apoBb.1MO_* = 13). (**D** and **E**) WISH with riboprobes against *prox1a* at 2 dpf (**D**) (*n*_WT_ = 4/4, *n_apoBa–/– apoBb.1MO_* = 7/9) and *tfa* at 4 dpf (**E**) (*n*_WT_ = 7/7, *n_apoBa–/– apoBb.1MO_* = 5/6) shows bilateral liver buds in *apoBa*^–/–^ *apoBb.1^MO^* embryos as opposed to *apoBa*^–/–^ mutants, which display a single liver bud located to the left of the midline. (**F**) Expression of forkhead box a2 (*foxa2*) at 2 dpf remains unchanged in *apoBa*^–/–^ *apoBb.1^MO^* embryos as compared with *apoBa*^–/–^ siblings. (**G**) Confocal images of 5 dpf *Tg(12xNRE:Egfp)* embryos with outlined livers, showing decreased Notch signal in the bile ducts of *apoBa*^–/–^ *apoBb.1^MO^* embryos (*n*_WT_ = 4, *n_apoBa–/–_* = 8, *n_apoBb1–/–_* = 6, *n_apoBa–/– apoBb.1MO_* = 20). (**H**) Confocal images of Nile red staining at 4 dpf depicting neutral lipid accumulation in *apoBb.1^MO^* and *apoBa*^–/–^ *apoBb.1^MO^* embryos. Red staining labels polar lipid; green fluorescence highlights neutral lipids (*n*_WT_ = 5, *n_apoBa–/–_* = 8, _napoBb.1–/–_** = 5, *n_apoBa–/– apoBb.1MO_* = 9). Scale bar: (**A**, **B**, **G**, and **H**) 50 μm, (**C**–**F**) 100 μm.

Next, we analyzed the development of the liver and the intestine, the main organs responsible for ApoB synthesis, assembly, and secretion in vertebrates (3). We took advantage of the fact that, in contrast to ApoB-null mice, *apoBa^–/–^ apoBb.1^–/–^* double mutants survive beyond the initial stages of formation of the digestive system to investigate the role of ApoB during normal formation of these organs. To examine liver morphology, we used *Tg(-2.8fabp10a:EGFP)^as3^*, a well-established transgenic reporter expressing EGFP under the regulation of the *fatty acid-binding protein 10A* (*fabp10a*) promoter (22). Injection of *apoBb.1* MOs into *Tg(fab10a:EGFP) apoBa^–/–^* embryos resulted in significantly reduced liver size at 4 dpf (Figure 2A and Supplemental Figure 3A), which was not due to apoptotic cell death, as confirmed by TUNEL staining (Figure 2B). Similar results were obtained following in situ hybridization for *fabp10a* (Figure 2C), suggesting a defect in hepatocyte specification, differentiation, and/or proliferation. To distinguish between these possibilities, we analyzed the expression of prospero homeobox 1a (*prox1a*), a transcription factor essential for hepatocyte differentiation and liver development (23, 24). As seen in Figure 2D, *prox1a*^+^ hepatic progenitors were normally detected to the left of the midline at 2 dpf, in phenotypically normal *apoBa^–/–^* embryos. By contrast, loss of both *apoBa* and *apoBb.1* caused embryos to develop bilateral livers (Figure 2D, arrowheads), suggesting an early role for ApoB in controlling liver laterality. This defective positioning of hepatoblasts was still detected at 4 dpf, as depicted by the expression of *transferrin* (*tfa*), an additional liver-specific marker (ref. 25 and Figure 2E). Interestingly, these defects were specific for the liver because we did not detect laterality problems in gut looping (Figure 2F, arrowheads, and Supplemental Figure 3B) or in early left-right asymmetry markers, such as *lefty1* (not shown). These results indicate that hepatoblast specification is initiated normally in *apoB*-mutant embryos but that liver formation begins to be affected after 2 days of development.

Liver organogenesis in zebrafish involves 2 main phases: first, specification and migration of *hhex^+^* and *prox1^+^* hepatoblasts to form the liver bud on the left side of the embryo, and second, hepatoblast differentiation into hepatocytes and biliary epithelial cells (BECs), followed by massive cell proliferation (26). To check whether the development of the intrahepatic biliary network is also affected in *apoB* mutants, we used the *Tg(EPV.Tp1-Mmu.Hbb:EGFP)^ia12^* reporter (12xNRE:EGFP), which consists of 12 repeats of Notch-responsive elements driving EGFP expression (27), because Notch signaling in hepatic progenitor cells was shown to be both required and sufficient for biliary specification (28). At 5 dpf, the *Tg(12xNRE:EGFP)* positive intrahepatic biliary network was highly branched in WT, *apoBa^–/–^*, and *apoBb.1^–/–^* larvae (Figure 2G), as opposed to *apoBa^–/–^ apoBb.1^MO^* that displayed largely reduced Notch activity. Taken together, the observed phenotypes suggest that, while ApoB is not required for initial hepatocyte specification, it appears to play an important role in hepatoblast positioning (i.e., budding and/or migration), proliferation, and segregation between hepatocytes and BECs.

Hepatic steatosis, the accumulation of lipid within hepatocytes, is a critical step in the pathogenesis of several human diseases, including alcoholic liver disease and NAFLD. While NAFLD is mostly associated with hyperlipidemia (29), defective synthesis and secretion of ApoB lipoproteins can also lead to hepatic steatosis, as observed in patients with FHBL1 (9, 30). To investigate whether the different *apoB* mutants develop steatosis, we utilized Nile red staining, which labels polar lipids in red and neutral lipids in green (31, 32). As expected, liver lipid stores were nearly undetected in WT and *apoBa^–/–^* mutants (Figure 2H), as opposed to *apoBb.1^MO^*, which featured excessive accumulation of lipid droplets in hepatocytes (Figure 2H). Interestingly, lipid droplet accumulation was less severe in the livers of *apoBa^–/–^* *apoBb.1^MO^* embryos (Figure 2H), most probably due to the impaired YSL lipoprotein production (Figure 2H, arrowheads). These results were confirmed by H&E staining of histological sections (Supplemental Figure 3C, arrowheads).

**Figure 3:**
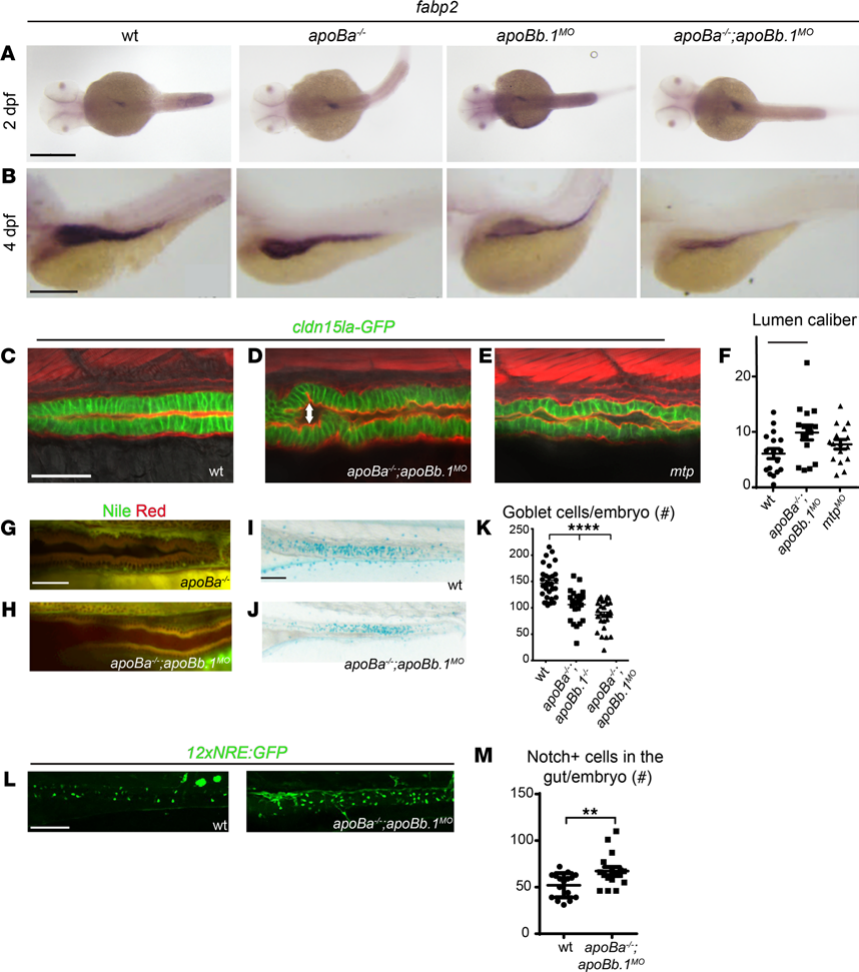
Gut development is impaired in *apoB* mutants. (**A** and **B**) WISH showing the spatial expression of *fabp2* at 2 (**A**) and 4 (**B**) dpf. (**C**–**E**) Confocal images at 4 dpf of WT (**C**), *apoBa*^–/–^ *apoBb.1^MO^* (**D**), and *mtp^MO^* (**E**) embryos in the *TgBAC(cldn15la-GFP)^pd1034^* reporter background, stained with phalloidin (red). Enlarged lumen caliber (arrow) is detected in *apoBa*^–/–^ *apoBb.1^MO^* and *mtp^MO^* embryos, as quantified (**F**) (*n* = 2, *n*_WT_ *=* 17, *n_apoBa–/– apoBb.1MO_ =* 15, *n_mtpMO_ =* 16). The data are shown as the mean ± SEM, calculated using ANOVA followed by Tukey’s multiple-comparison test. *P < 0.05. (**G** and **H**) Confocal images of the guts of 4 dpf *apoBa^–/–^* and *apoBa*^–/–^ *apoBb.1^MO^* embryos stained with Nile red, show no lipid accumulation. (**I** and **J**) Bright-field images of 5 dpf embryos stained with Alcian blue showing reduced number of goblet cells in *apoBa*^–/–^ *apoBb.1*^–/–^ double mutants (**J**) as compared with WT embryos (**I**), as quantified (**K**) (*n* *=* 3, *n*_WT_ *=* 30, *n_apoBa–/– apoBb.1–/–_ =* 27, *n_apoBa–/– apoBb.1MO_ =* 27). The data are shown as the mean ± SEM, calculated using ANOVA followed by Tukey’s multiple-comparison test. ****P < 0.0001. (**L**) Confocal images at 5 dpf of WT and *apoBa*^–/–^ *apoBb.1^MO^* embryos in the *Tg(12xNRE:Egfp)* reporter background. (**M**) Quantification of number of cells displaying active Notch signaling in the guts of WT and *apoBa*^–/–^ *apoBb.1^MO^* embryos. The data are shown as the mean ± SEM, calculated using 2-tailed Student’s *t* test. Scale bar: (**A**–**E**, **G**, and **H**) 50 μm, (**I**, **J**, and **L**) 100 μm. **P < 0.01. *P* *<* 0.05 (considered significant versus control group).

Because of the close relationship between liver and intestine formation, we tested whether ApoB depletion also affects gut development. The intestine is responsible for the absorption of lipids and their secretion in the form of chylomicrons into lymphatic vessels. Analysis of *foxa2* (ref. 33; Figure 2F, arrowheads; and Supplemental Figure 3B) and fatty acid binding protein 2 (*fabp2*) (ref. 25 and Figure 3A) expression revealed normal development of the intestinal tube in individual mutants at 2 dpf. However, by 4 dpf we noticed that the gut appeared somewhat reduced in size in *apoBa^–/–^ apoBb.1^MO^* embryos (Figure 3B). Yet, the effects of ApoB depletion on gut formation appeared to be less severe than the effects observed in the liver. We also inspected the guts of the different mutants by mating the fish with the *TgBAC(cldn15la-GFP)^pd1034^* (34) reporter, which labels tight junctions in the intestine. We detected slightly enlarged lumens in the guts of *apoBa^–/–^ apoBb.1^MO^* embryos (Figure 3, C, D, and F) as compared with WT siblings. Similar effects were observed following injection of *mtp* MOs (Figure 3, C, E, F). These defects were corroborated by direct inspection of H&E staining of histological sections (Supplemental Figure 3D). Although at this stage of development the intestine is known to be folded and to contain polarized epithelial cells with a microvillus brush border (ref. 35 and Supplemental Figure 3D), in *apoBa^–/–^ apoBb.1^–/–^* embryos, the intestinal epithelium appeared thin and poorly folded, and the cells had only few scattered microvilli as compared with their WT counterparts (Supplemental Figure 3, D and E, blue arrowheads). Also, intestine-specific deletion of *Mtp* or *ApoB* in mice renders small and large intestines with enlarged calibers (36, 37), mostly due to fat accumulation in the villus enterocytes, fully recapitulating the phenotypes reported for abetalipoproteinemia (38). In contrast, we did not detect lipid accumulation in enterocytes of *apoBa^–/–^ apoBb.1^–/–^* mutants (Figure 3, G and H), suggesting that the enlarged lumen phenotype and thinner epithelia are most likely derived from impaired nutrient absorption, as previously described (39).

Finally, Alcian blue staining at 5 dpf highlighted a significantly reduced number of goblet cells in *apoBa^–/–^ apoBb.1^–/–^* mutant guts (Figure 3, I–K). Previous reports have shown that inhibition of Notch signaling directs the specification of intestinal cells toward a secretory fate (40), leading to increased numbers of goblet cells. We therefore evaluated the levels of Notch activation in the guts of WT and mutant embryos using the 12NRE:GFP reporter. As seen in Figure 3, L and M, we detected increased numbers of Notch^+^ cells in the guts of *apoBa^–/–^ apoBb.1^MO^* embryos as compared with WT siblings, pointing to alteration in Notch signaling as the mechanism underlying goblet cell differentiation. Altogether, our results indicate that ApoB deficiency results in impaired liver development accompanied by hepatic steatosis and disrupted intestinal architecture.

### ApoB depletion affects vascular development.

**Figure 4:**
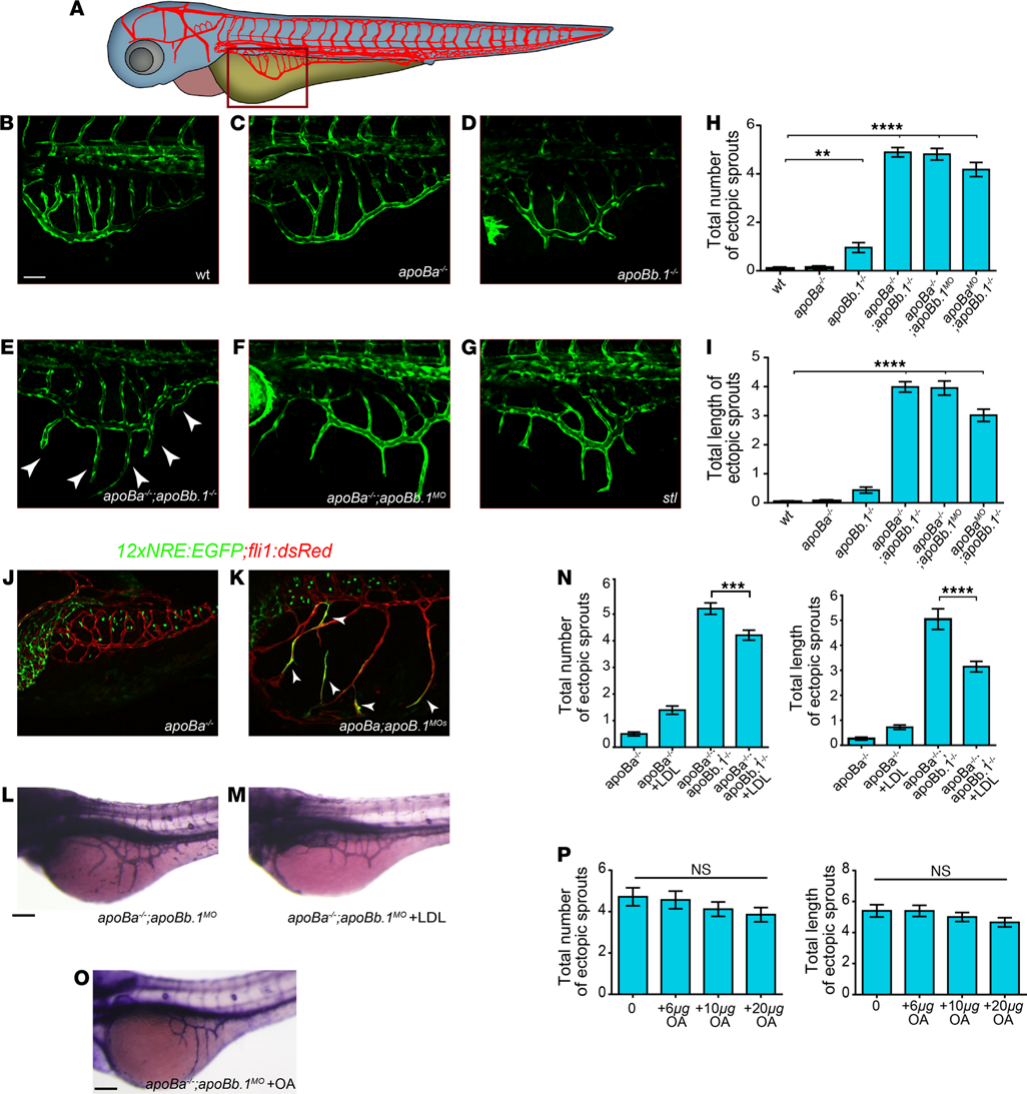
*apoB* mutants display hyperangiogenic phenotypes. (**A**) Schematic representation of the zebrafish embryonic vasculature, with red square marking the subintestinal vessels (SIVs). (**B**–**G**) Confocal images at 3 dpf showing ectopic sprouts (arrowheads) arising in the SIVs of *apoba*^–/–^ *apoBb.1*^–/–^ double mutants (**E**), and *apoba*^–/–^ *apoBb.1^MO^* (**F**) and stalactite (*stl*) mutants (**G**), but not in WT (**B**), *apoBa*^–/–^ (**C**), or *apoBb.1*^–/–^ (**D**) animals. (**H** and **I**) Quantification of the number (**H**) and length (**I**) of ectopic sprouts at 3 dpf. *n* *=* 3, *n*_WT_ *=* 45, *n_apoBa–/–_ =* 55, *n_apoBb.1–/–_ =* 23, *n_apoBa–/– apoBb.1–/–_ =* 31, *n_apoBa–/– apoBb.1MO_ =* 40, *n_apoBb.1–/– apoBaMO_ =* 21. The data are shown as the mean ± SEM, calculated using ANOVA followed by Tukey’s multiple-comparison test. ****P < 0.0001, **P < 0.01. (**J** and **K**) Confocal images at 5 dpf of *apoBa^–/–^* and *apoba*^–/–^ *apoBb.1^MO^* embryos in the *Tg(12xNRE:Egfp)* reporter background. White arrowheads in *apoBa^–/–^ apoBb.1^MO^* embryos point to endothelial cells (ECs) with active Notch signaling, in ectopic sprouts that failed to retract. *n* *=* 2, *n_apoBa–/–_ =* 10, *n_apoBa–/– apoBb.1MO_ =* 12. (**L** and **M**) Alkaline phosphatase (AP) staining of the SIVs at 3 dpf, showing inhibition of ectopic sprouting following intravascular injection of DiI-LDL into *apoBa^–/–^ apoBb.1^MO^*. (**N**) Quantification of number and length of ectopic sprouts following intravascular injection of DiI-LDL (*n* *=* 3, *n_apoBa–/–_ =* 57, *n*_apoBa–/–+LDL_ *=* 43, *n_apoBa–/– apoBb.1MO_ =* 67, *n*_apoBa–/– apoBb.1MO+LDL_ *=* 57). The data are shown as the mean ± SEM, calculated using ANOVA followed by Tukey’s multiple-comparison test. ****P < 0.0001, ***P < 0.001. (**O**) AP staining of the SIVs of 3 dpf *apoBa^–/–^ apoBb.1^MO^* embryos treated with OA, as quantified (**P**) (*n* *=* 3, *n_apoBa–/– apoBb.1MO_ =* 20, *n_apoBa–/– apoBb.1MO+6μg__OA_ =* 19, *n_apoBa–/– apoBb.1MO+10μg__OA_* = 21, *n*_apoBa–/– apoBb.1MO+20μg_ *_OA_* = 22). The data are shown as the mean ± SEM, calculated using ANOVA followed by Tukey’s multiple-comparison test. Scale bar: (**B**–**G**) 50 μm, (**J**–**O**) 100 μm. *P* *<* 0.05 (considered significant versus control group).

Previous studies, including our own, have demonstrated that ApoB-containing lipoproteins have an inhibitory effect on vascular growth (2, 15, 16, 41). We therefore decided to analyze the involvement of the ApoB isoforms in the development of the vascular system by assessing the early vasculature of the different mutants. We first focused on the subintestinal vessels (SIVs), a plexus located on top of the YSL (Figure 4A) that plays a critical role in yolk absorption and vascularization of the gastrointestinal tract (15, 27, 42). In line with the lipid and morphological defects, the vascular phenotypes were more pronounced in the double homozygous mutants, as compared with WT siblings or single mutants. As seen in Figure 4, B–D and H–I, the SIVs formed normally in *apoBa^–/–^* and displayed only minor defects in *apoBb.1* mutants. By contrast, *apoBa* *apoBb.1* double mutants (Figure 4, E, H, and I) and *apoBa^–/–^ apoBb.1^MO^* (Figure 4, F, H, and I) larvae exhibited ectopic angiogenic sprouts arising in the SIVs, which did not retract during the remodeling phase as they did in WT siblings (27). These sprouts, reminiscent of those observed in *stl* mutants (Figure 4G and ref. 15), were clearly detected following injection of *apoBb.1* MOs into WT embryos (Figure 4, E, F, H, and I). However, no effects were observed upon injection of *apoBb.1* MOs into *apoBb.1* mutants (Supplemental Figure 4, B–E), confirming the MO specificity. Similar hyperangiogenic behaviors were detected in the posterior cerebral vein (PCeV) at 4 dpf (Supplemental Figure 4, A and F–H), and in the dorsal trunk (Supplemental Figure 4, A and I–K) of *apoBa^–/–^ apoBb.1^MO^* larvae, suggesting that impaired angiogenesis is a general consequence of ApoB depletion that becomes more pronounced as development proceeds.

We have previously shown that ApoB lipoproteins induce ectopic angiogenesis by downregulating the expression of the VEGF decoy receptor Vegfr1/Flt1 (15). Accordingly, depletion of both membranous and soluble forms of Flt1 fully phenocopied this phenotype (27). Given that loss of Flt1 has been shown to result in enhanced Notch signaling (43), and that Flt1^−/−^ mutant sprouts are less likely to retract (44), we decided to investigate whether this mechanism is activated in *apoB* mutants. Live imaging of the *12xNRE:EGFP* reporter revealed clear activation of Notch signaling in the ectopic sprouts that failed to retract in *apoBa^–/–^ apoBb.1^MO^* embryos (Figure 4, J and K). In contrast, no ECs displaying active Notch signaling were detected in the SIV plexus of WT embryos at comparable developmental stages (i.e., 5 dpf), when the vessels have acquired their final, quiescent stage. Taken together, these results reinforce and expand our previous findings by showing that not only Flt1 but also Notch signaling act downstream of ApoB to modulate angiogenesis.

In order to verify that the observed vascular phenotypes indeed derive from the absence of ApoB lipoproteins, we attempted to rescue the defects by intravascularly injecting DiI-labeled LDL into *apoBa^–/–^ apoBb.1^MO^* embryos (Supplemental Figure 4L) as previously described (15, 17). As seen in Figure 4, L–N, a single injection of DiI-LDL was sufficient to revert the excessive angiogenesis phenotype, as opposed to oleic acid (OA) supply (Figure 4, O and P), supporting a specific role for ApoB, and not the lipid moieties within lipoproteins, in developmental angiogenesis. These results are in line with our previous findings, which showed that the strong excessive angiogenesis phenotype *stl* mutants displayed in response to lipoprotein depletion (Figure 4G) was efficiently reversed following injection of a delipidated form of ApoB (15).

**Figure 5:**
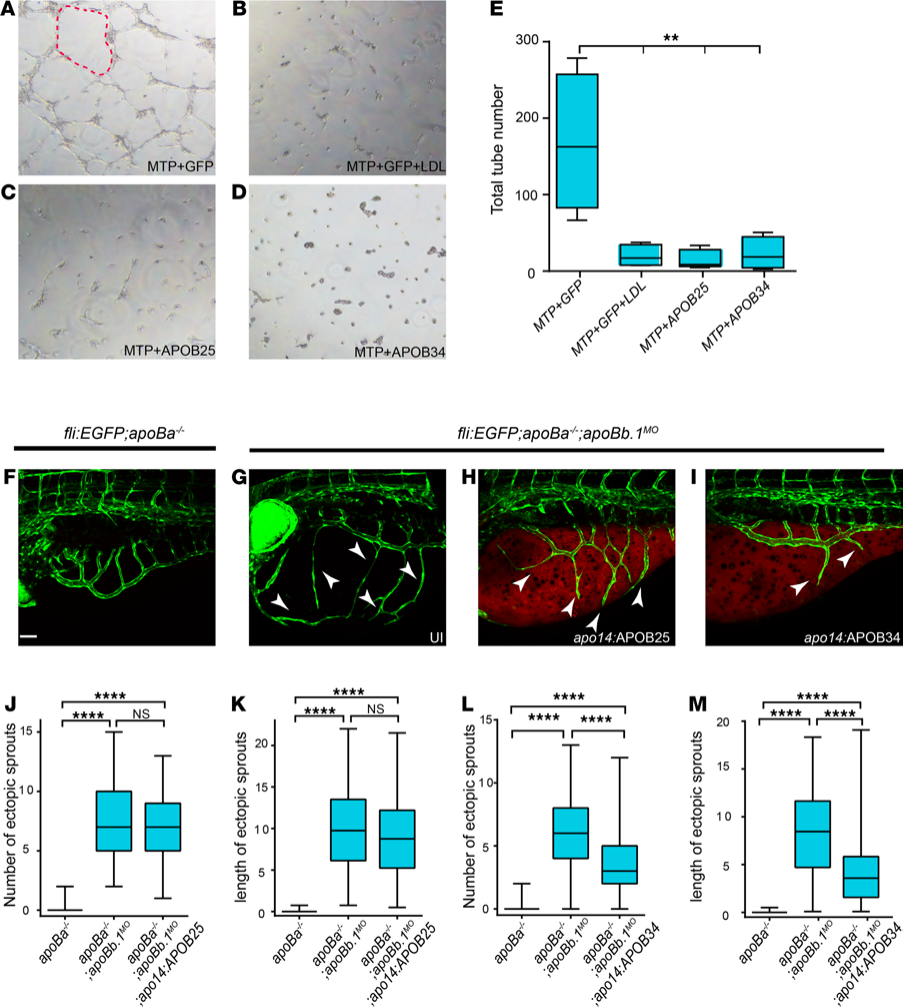
Truncated forms of human APOB inhibit angiogenesis in *apoB* mutants. (**A**–**D**) Tube formation assay on HUVECs untreated (**A**, **C**, and **D**) or treated with LDL (**B**) plus conditioned medium from HEK293 cells cotransfected with (**A** and **B**) *MTP+GFP*, (**C**) *MTP+APOB25*, and (**D**) *MTP+APOB34*. Red dashed line outlines vascular tube structure. (**E**) Quantification of total number of tubes following different treatments (*n* *=* 3, *n_MTP+GFP_ =* 4, *n_MTP+GFP+LDL_ =* 4, *n_MTP+APOB25_ =* 5, *n_MTP+APOB34_ =* 4). The data are shown as the mean ± SEM, calculated using ANOVA followed by Tukey’s multiple-comparison test. **P < 0.01. (**F**–**I**) Confocal images at 4 dpf showing reduction in the number and length of ectopic sprouts in *Tg(fli1:EGFP) apoBa^–/–^ apoBb.1^MO^* embryos injected with *apo14:APOB34* (**I**) as compared with uninjected *apoBa^–/–^* (**F**), uninjected *apoBa^–/–^ apoBb.1^MO^* (**G**), and *apoBa^–/–^ apoBb.1^MO^* injected with *apo14:APOB25* (**H**). White arrowheads point to ectopic sprouts. (**J**–**M**) Quantification of the number and length of ectopic sprouts in *apo14 APOB25* (**J** and **K**) (*n* *=* 3, *n_apoBa–/–_ =* 47, *n_apoBa–/– apoBb.1MO_ =* 54, *n_apoBa–/– apoBb.1MO apo14:APOB34_ =* 54) and *apo14 APOB34* (**L** and **M**) (*n* *=* 3, *n_apoBa–/–_ =* 47, *n_apoBa–/– apoBb.1MO_ =* 51, *n_apoBa–/– apoBb.1MO apo14:APOB34_ =* 55) injected embryos. The data are shown as the mean ± SEM, calculated using ANOVA followed by Tukey’s multiple-comparison test. Scale bar (**F**–**I**): 50 μm. ****P < 0.0001. *P* *<* 0.05 (considered significant versus control group).

Because this pathway could potentially be exploited to control pathological neovascularization, we proceeded to define the domains within ApoB that act on ECs. To this end, we tested the ability of truncated forms of the human APOB protein to inhibit angiogenesis, using the well-established tube formation assay. To ensure that lipoproteins are presented to the cells in their physiological form, we cotransfected MTP along with human APOB34 or APOB25, which have been previously shown to undergo proper lipidation and secretion (45, 46), into HEK293 cells (Supplemental Figure 4M). We then transferred the media containing the different truncated forms to human umbilical vein endothelial cells (HUVECs) and examined their ability to generate tubes. LDL, which we previously showed inhibits tube formation in culture (16), was used as positive control. Interestingly, both APOB25 and APOB34 forms inhibited angiogenesis in this assay, suggesting that the antiangiogenic effect of ApoB is conveyed by the 25% (N-terminus) of the protein (Figure 5, A–E).

We then asked whether these fragments are sufficient to inhibit angiogenesis in vivo. To answer this question, we generated plasmids expressing human *APOB25* and *APOB34* fused to *IRESmCherry*, under the regulation of the *apo14* promoter, which drives expression in the YSL and the liver (47). While the hyperangiogenic phenotype of *apoBa^–/–^ apoBb.1^MO^* embryos was not reverted following *apo14:APOB25-IRESmCherry* injection (Figure 5, F–H, J, and K), *apoBa^–/–^ apoBb.1^MO^* embryos injected with *apo14:APOB34-IRESmCherry* displayed a significant reduction of both the number and length of the ectopic sprouts at 4 dpf (Figure 5, G, I, L, and M). These results suggest that the first 34% of the ApoB protein is required to exert its antiangiogenic effect in vivo. The discrepancy between the in vivo and the in vitro results may derive from differences in the mobility and clearance of the truncated forms of APOB in vivo. Specifically, it has been shown that APOB25 is not present in circulation in vivo, despite it being produced and secreted into the blood (45, 48), due to fast clearance.

As a whole these results demonstrate the applicability of our newly generated models of ApoB deficiency for assessing the physiological activity of potential therapeutic agents.

## Discussion

In this work, we generated zebrafish mutants for 2 ApoB isoforms: *apoBa* and *apoBb.1*. Similar to *ApoB*-null mice, zebrafish carrying mutations in both *apoBa* and *apoBb.1* demonstrate early lethality. However, in contrast to the mammalian models, the external fertilization and development of zebrafish allowed us to investigate previously unappreciated roles of ApoB during embryogenesis.

Three *apoB* genes are present in the zebrafish genome, namely *apoBa*, *apoBb.1*, and *apoBb.2*. During early development, *apoBa* and *apoBb.1* are highly expressed, whereas *apoBb.2* is absent (14). Interestingly, despite the strong expression of *apoBa* mRNA, its protein levels are almost 20-fold lower than those of ApoBb.1 (14). Accordingly, while the absence of *apoBa* did not elicit any noticeable phenotypes, *apoBb.1* depletion caused strong hypolipidemia. Finally, deletion of both isoforms resulted in severe phenotypes and lethality.

Our results uncover the roles for ApoB during development of the liver, one of its main producing organs. In particular, we find that *apoBa^–/–^ apoBb.1^MO^* embryos display similar phenotypes to those caused by retinoic acid (RA) deficiency (49), including decreased liver volumes and liver bud bilaterality. Zebrafish embryos treated with the Raldh inhibitor DEAB, or injected with MOs against the 3 RA receptors (*rarab*, *raraa*, and *rarga*), were shown to display smaller livers because of impaired hepatocyte proliferation. In contrast, RA addition to the fish water resulted in massive proliferation of hepatocytes and enlarged livers (49). In addition, downregulation of the RA receptor *rargb* led to bilateral liver buds and intrahepatic biliary defects, similar to those displayed by double *apoB* mutants. While most RA circulation is mediated through retinol binding protein 4, it has been shown that ApoB-containing lipoproteins (e.g., LDL and VLDL) also participate in RA shuttling (50). Therefore, the possibility exists that complete depletion of ApoB leads to RA unavailability, which in turn results in liver bilaterality and reduced hepatocyte and BEC proliferation.

The absence of both apoB isoforms resulted in low levels of TGs and cholesterol in circulation, accompanied by massive lipid droplet accumulation in hepatocytes, and liver steatosis. These phenotypes, reminiscent of human NAFLD, recapitulate part of the clinical manifestations of FHBL1, making the *apoBa^–/–^ apoBb.1^–/–^* mutant zebrafish an advantageous model for the study of this disorder and for the identification of potential treatments.

In addition to liver abnormalities, ApoB and MTP deficiency resulted in disrupted intestinal architecture. In particular, the guts were poorly folded and displayed enlarged lumen calibers, resembling phenotypes displayed by starved WT larvae (39, 51). Double *apoB* mutants feature an increased number of goblet cells. Interestingly, it has been shown that RA negatively regulates goblet cell differentiation (52), whereas inhibition of Notch signaling directs specification of intestinal cells toward a secretory fate (40) leading to increased numbers of goblet cells. Our results indeed demonstrate increased activation of Notch signaling in the intestines of *apoBa^–/–^ apoBb.1^–/–^* mutants, supporting the idea that ApoB affects goblet cell differentiation through activation of Notch targets. Downstream of Notch, the transcription factor Kruppel-like factor 4 (Klf4) has been shown to be required for terminal differentiation of goblet cells (53). Expression of *klf4a* was shown to be controlled by Notch signaling (i.e., embryos treated with the γ-secretase inhibitor DAPT display increased *klf4a* expression in the intestine, while decreased *klf4a* expression and reduction in goblet cell number were observed in embryos injected with *Notch intracellular domain* mRNA; ref. 54). On the other hand, it has been demonstrated that the RA/RARa axis negatively modulates *klf4* expression, thereby impacting goblet cell differentiation as well. In the future it will be interesting to investigate whether ApoB is involved in the interplay between the Notch and RA/RARa pathways in the developing intestine.

Finally, the phenotypes detected in the liver and intestine of *apoB* mutants could result from the absence of fat-soluble vitamins, such as vitamins E and A, and their RA metabolites, whose absorption and transport throughout the body rely mostly on ApoB, in the form of chylomicrons (55, 56).

Previous studies have demonstrated the strong impact of lipoproteins on EC behavior (15, 17, 57, 58); yet, the mechanisms by which ApoB affects these cells are not fully understood. The double *apoB* mutant exhibited a marked angiogenic phenotype characterized by excessive sprouts in the SIVs, PCeV, and arterial intersomitic vessels. This angiogenic effect may result from a combination of factors related to ApoB deficiency, such as lipid raft density, lack of fat-soluble vitamins, and activation of downstream signaling pathways in ECs. Interestingly, the angiogenic defects were observed only following complete depletion of circulating ApoB lipoproteins. Although *apoBb*.*1* mutants featured severe hypolipidemia, they did not display a strong angiogenic phenotype, suggesting that small amounts of ApoB are sufficient to maintain healthy control over the angiogenic process and further confirming the specific effects of the ApoB protein on EC behavior.

Our structure-function analyses revealed that APOB34, a truncated form of human APOB lacking the LDL receptor binding domain, is able to rescue the angiogenic phenotype in double *apoB* mutants. These results raise the hypothesis that an LDL receptor–independent pathway might regulate the effects of ApoB on ECs. Recently, it has been shown that LDL can bind the TGF-β receptor activin receptor-like kinase 1 (ALK1; ref. 59), which is also involved in lipoproteins’ transcytosis (60). Moreover, ALK1 has been shown to exert antiangiogenic effects via upregulation of VEGFR1 (61). Interestingly, *stl* mutants display reduced expression of *vegfr1*, leading to excessive angiogenesis (15). Therefore, it seems feasible that APOB induction of ALK1 activity leads to upregulation of *vegfr1* and inhibition of angiogenesis.

Overall, our newly generated *apoB* mutants can serve as a model for studying human pathologies associated with hypolipidemia, as well as for related drug screens. Moreover, understanding the molecular mechanisms by which ApoB regulates angiogenesis will provide potential new therapeutic targets for the treatment of vascular pathologies ranging from cancer to ischemic heart disease.

## Methods

### Zebrafish husbandry and transgenic lines.

*Tg(fli1:EGFP)^yl^*, *stl* (15), *TgBAC(cldn15la-GFP)^pd1034^* (34), *Tg(fli1:dsRed)^um13^* (62), T*g(lyve1:dsRed2)^nz101^* (63), *Tg(flt1_9a_cFos:GFP)^wz2^* (63), *Tg(EPV.Tp1-Mmu.Hbb:EGFP)^ia12^* (27), and *Tg(-2.8fabp10a:EGFP)^as3^* (22) have been previously described.

To generate *apo14:APOB25-IRESmCherry* and *apo14:APOB34-IRESmCherry*, the zebrafish *apo14* promoter (47) was cloned into pDONRP4-P1R (Invitrogen, Thermo Fisher Scientific), human *APOB25* and *APOB34* (45) were cloned into pDONR221 (Invitrogen, Thermo Fisher Scientific), and the *IRESmCherry* sequence was cloned into pDONRP2R-P3 (Invitrogen, Thermo Fisher Scientific), using Gateway BP Clonase II (Invitrogen, Thermo Fisher Scientific, 11789-020). The 3 vectors were then transferred into pDestTol2pA2 (62) using a Gateway LR reaction (Invitrogen, Thermo Fisher Scientific, 12538-120). The plasmids were injected along with *Tol2 transposase* mRNA into 1-cell-stage embryos (62).

### Generation of zebrafish mutants.

The *apoBb.1* CRISPR guide was designed with CHOPCHOP; potential off-target sequences were assessed using the MIT CRISPR Design site. Oligonucleotides synthesized for the guide sequence GGACTAGTGTGGTCTTTGAC were cloned into the BsmBI sites of the pT7-gRNA plasmid (Addgene plasmid 46759). *Cas9* mRNA was generated from pCS2-nCas9n (64) using mMACHINE T7 ULTRA kit (Ambion, AM1345). *Cas9* mRNA (250 ng/μL) and guide RNAs (100 ng/μL) were coinjected into 1-cell-stage *Tg(fli1:EGFP)^y1^* transgenic embryos. For genotyping, genomic DNA was extracted at 24 hours postfertilization (hpf), amplified with 5′-TACTCTGTGAATGCCAGACAGG and 5′-TCAGAAGCTATCCAGCAAACA (184 bp) primers, and analyzed on a 3% agarose gel. *apoBa* TALENs left — TACAGCTAACCTCAAGAA — and right — TGCCAGgtacaaaaaca plasmids were generated as described (65) and transcribed using mMESSAGE mMACHINE T7 ULTRA kit (Ambion, AM1345). mRNAs (250 ng each) were coinjected into 1-cell stage. For genotyping, genomic DNA was extracted at 24 hpf, amplified with 5′-TATCAGTACACAGCAGAGAGCA and 5′-TCGTAAAATTAGGCTAAGCCA (110 bp), and analyzed on a 3% agarose gel.

### Antisense morpholino injection.

The following antisense MOs (Gene Tools) were resuspended and injected as described (66) at the following concentrations: *mtp* (15) (4 ng), *apoBa* (15) (4 ng), and *apoBb.1* (CCATGATGGGTTCAGGTAAGCTCGT) (2 ng).

### In situ hybridization and staining procedures.

Embryos were fixed overnight in 4% paraformaldehyde (PFA) and processed for ORO (15), AP (15), Nile red (MilliporeSigma, N3013; refs. 31, 32), and Alcian blue (MilliporeSigma, A3157; ref. 67) staining, as described. TUNEL staining was performed using *In Situ* Cell Death Detection Kit, TMR red (Roche, 12156792910), following manufacturer’s instructions.

In situ hybridization was performed as described (68) using the following probes: *fabp10a* (69) and *prox1a* (70). The following primers were used to generate the corresponding riboprobes: *apoBa* 5′-GACCTTGGCTTTCCGTTCC-3′, 5′-CAGCAGGGAAGCTCTCTATGAA-3′; *apoBb*.*1* 5′-GCTGCAGTGTATGCCATGGGAAT-3′, 5′-ATCAACAGTGGGTTCCAGACCCTT-3′; *foxa2* 5′-GTGTTACACCTCGGTCAGCA-3′, 5′-CACTTGAAGCGCTTTTGCCT-3′; *fabp2* 5′-TGGAAAGTCGACCGCAATGAGA-3′, 5′-TACCTTTCCGTTGTCCTTGCGT-3′; *tfa* 5′-TCTGGAGGCTGGAATACTCCTA-3′, 5′-TAAACCTGAGCCCTTACGCA-3′.

### Assessment of vascular phenotypes.

Larvae were assayed for SIV ectopic sprouting using fluorescence imaging (PCeV, trunk) or AP staining (SIVs). Quantitation of the SIVs’ phenotype was done using a grid lens in a Leica stereoscope under ×10 original magnification; each side of the animal was scored separately.

### Total RNA isolation and semiquantitative reverse transcription PCR.

A pool of 10–20 embryos/sample was homogenized in TRIzol (Invitrogen, Thermo Fisher Scientific, 15596026) and processed for RNA extraction following standard procedures (15). A total of 1 μg of RNA per reaction was reverse-transcribed using a High-Capacity cDNA Reverse Transcription Kit (Applied Biosystems, Thermo Fisher Scientific, 4368814). To measure relative changes in mRNA transcripts, we used the following primers: *apoBa* 5′-GACCTTGGCTTTCCGTTCC-3′, 5′-CAGCAGGGAAGCTCTCTATGAA-3′; *apoBb.1* 5′-GCTGCAGTGTATGCCATGGGAAT-3′, 5′-ATCAACAGTGGGTTCCAGACCCTT-3′; *apoBb.2* 5′-GTTCATAGGAGCGAGCATTGACCA-3′, 5′-AGACCCAAACTGTCAACGAAAGGC-3′. Expression levels were standardized to the primer set specific for β-actin 5′-TGACAGGATGCAGAAGGAGA-3′ and 5′-GCCTCCGATCCAGACAGAGT-3′.

### TG and cholesterol measurements.

Twenty deyolked embryos at 3 dpf per sample were gently homogenized in PBS with a pestle. After centrifugation at 15,000*g* for 15 minutes, supernatants were collected, and TG and cholesterol levels were measured using kits from BioVision (Triglyceride Quantification Kit, K622-100; Cholesterol Quantification Kit, K623-100) as described (71). For each genotype, 3 repeats of 10–20 embryos each were performed.

### Protein extraction and Western blot.

Zebrafish embryos at 3 dpf were processed for Western blot as described (70). Briefly, proteins were separated by SDS-PAGE using a 6% separating gel and a 4% stacking gel. Transfer was performed at 400 mA for 1 hour. Membranes were blocked with 2% BSA in PBS-Tween. The following antibodies were used: rabbit anti–human APOB100 (Abcam, ab20737), 1:1500; mouse monoclonal anti–human α-tubulin (MilliporeSigma, T5168), 1:3000.

### OA and LDL treatments.

OA (MilliporeSigma, O7501) was dissolved in ethanol and added to the fish water with fatty acid–free BSA (MilliporeSigma, A8806), at 6 to 20 μg/mL concentration.

Dil-LDL (Invitrogen, Thermo Fisher Scientific, L3482) was injected intravascularly at 2 dpf, as described (15).

### Imaging.

Confocal imaging was performed using a Zeiss LSM 780 upright confocal microscope (Carl Zeiss) with a W-Plan Apochromat ×20 objective, NA 1.0. Images were processed using ImageJ (NIH). Fluorescent proteins were excited sequentially with 488 nm and 563 nm single-photon lasers.

### Cell transfection and culture.

HEK293 cells (2) that were 50% confluent were cotransfected with 5 μg *Huh7MTP* (15) and 10 μg pGFP or *APOB34-FLAG* or *APOB25-DsRed* (45), using JET PEI (Polyplus, catalog 15021C1T) standard protocol. Twenty-four hours after transfection, medium was replaced with EBM-2 (endothelial basal medium) (Lonza, CC-3156), containing 0.5% lipoprotein-depleted serum (2) and 1% penicillin-streptomycin. Forty-eight hours later the medium was collected and centrifuged at 2000 rpm for 5 minutes to ensure a cell-free medium. The upper phase was used as conditional medium for the tube formation assay.

### Tube formation assay.

HUVECs (Lonza) were cultured on gelatin-coated dishes, in M199 medium supplemented with 20% FCS, 50 μg/mL Endothelial Cell Growth Supplement (Zotal catalog BT-203), 5 U/mL heparin, 1% penicillin-streptomycin, 2 mM l-glutamine (16).

For treatment, conditional medium collected from *MTP*+*GFP*, *MTP*+*GFP*+LDL (100 μg/mL) (BT-903), *MTP+APOB25*, and *MTP+APOB34* transfected HEK293 cells was added. Following 18-hour incubation, 30,000 cells were seeded on Matrigel (BD, 356231) for 8 hours and cultured with the appropriate conditional medium (*MTP*+GFP, *MTP+*GFP+LDL, *MTP+APOB25*, and *MTP+APOB34*). Cells were then fixed in 4% PFA and imaged using a bright-field microscope. Nine fields/well were acquired per experiment. Total tube numbers were quantified using ImageJ. For each experiment, values were normalized to the nontreated well.

### Statistics.

All data are reported as mean values ± SEM and were analyzed using Prism 5 software (GraphPad Software). Comparison of 2 samples was done by unpaired 2-tailed Student’s *t* test. Statistical significance for 3 or more samples was calculated via 1-way ANOVA followed by post hoc Tukey’s for multiple comparisons. A *P* value less than 0.05 was considered significant. In box-and-whisker plots, box indicates 25th to 75th percentiles, line indicates median, and whiskers represent min and max.

### Study approval.

Zebrafish were raised by standard methods and handled according to, and following approval by, the Weizmann Institute Animal Care and Use Committee (68).

## Author contributions

HT designed and conducted all experiments, analyzed data, and cowrote the manuscript; IAD conducted HUVEC experiments; NM managed fish work and conducted zebrafish experiments; and KY initiated and directed the study, designed experiments, analyzed data, and cowrote the paper with input from all authors.

## Supplementary Material

Supplemental data

## Version 1 07/08/2021

Electronic publication
