# Supplementary material for: Zebrafish mutants provide insights into Apolipoprotein B functions during embryonic development and pathological conditions
Source: JCI Insight. 2021 Jul 8;6(13):e130399. doi: 10.1172/jci.insight.130399 (PMC8410079; doi:10.1172/jci.insight.130399)
Supplement: Supplemental data [file jciinsight-6-130399-s022.pdf]

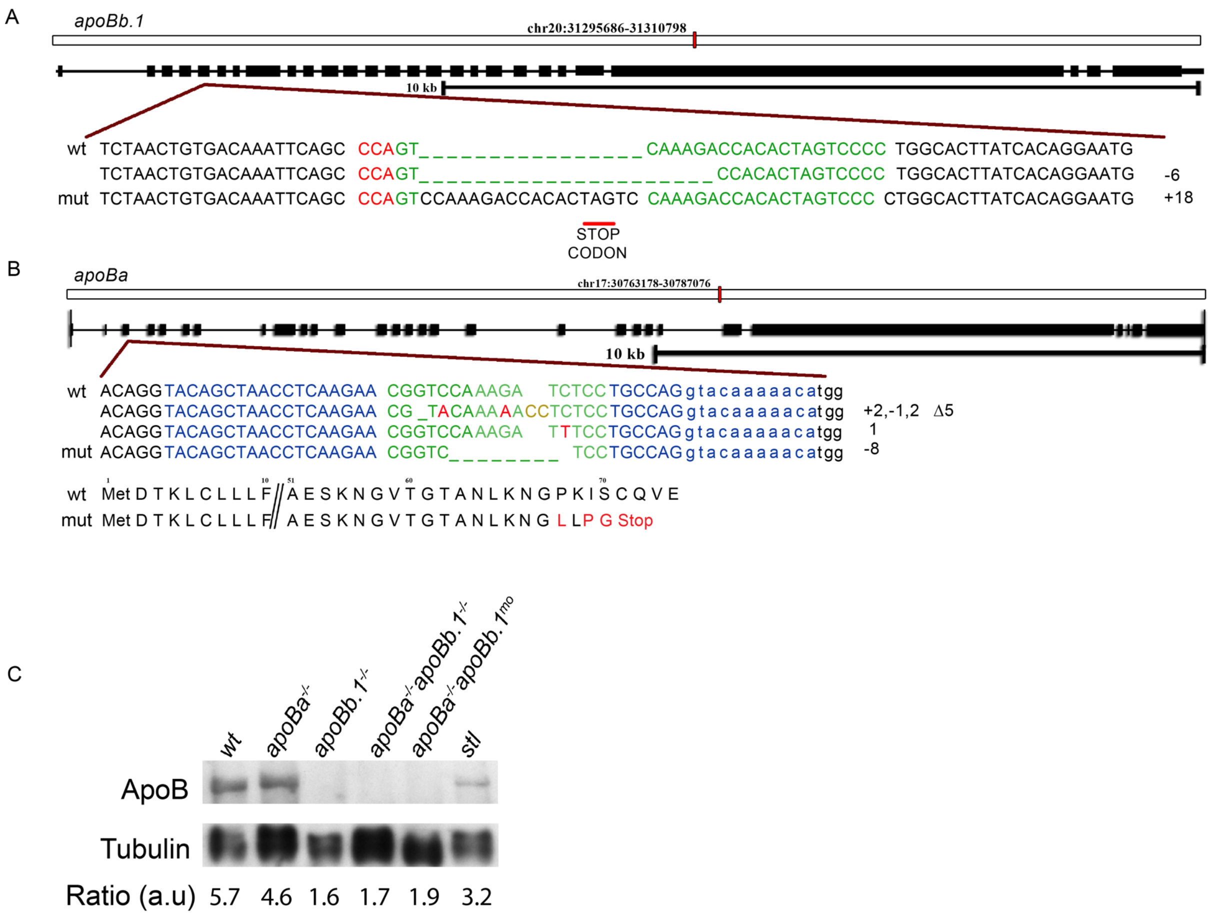

Supplemental Figure 1: Generation of *ApoB zebrafish mutants.* (A) Schematic representation of the *apoBb.1* genomic locus, with the guide target site in exon 5. Guide recognition site is shown in green and the PAM in red. The mutant sequence carries an 18bp insertion containing an in frame stop-codon (TAG). **(B)** *apoBa* genomic locus, with the putative TALEN site in exon 3. The sequence contains TALENs recognition sites (blue), and a TALEN spacer (16 nucleotides, green) with the 8-bp deletion in the mutant. **(C)** Western blot analysis showing complete depletion of the ApoB protein in *apoBb.1*$^{-/-}$, *apoBa;apoBb.1*$^{-/-}$ and *apoBa;apoBb.1*^{MO} animals.

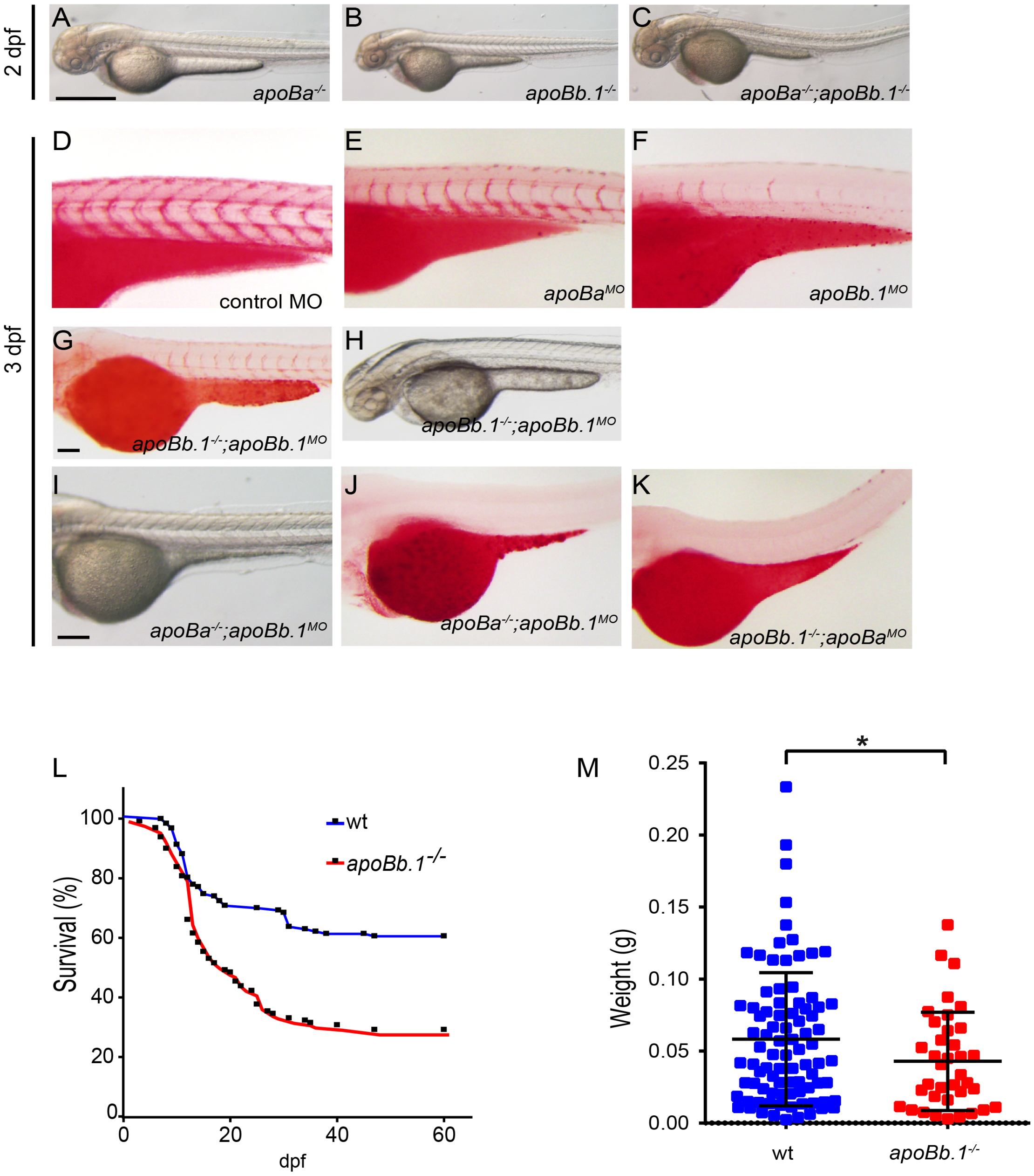

Supplemental Figure 2: (A-C) Bright field images of 2 dpf *apoBa*$^{-/-}$ (A), *apoBb.1*$^{-/-}$ (B) and *apoba*$^{-/-}$ *;apoBb.1*$^{-/-}$ (C) mutant embryos. **(D-F)** 3 dpf embryos injected with *apoBb.1* MO (F) show decreased lipid levels, as compared to embryos injected with control MO (D) OR *apoBa* MO (E). **(G,H)** Bright field image and ORO staining of 3 dpf *apoBb.1*$^{-/-}$ mutant injected with *apoBb.1* MO. **(I-K)** *apoBa* mutants injected with *apoBb.1* MO (I,J) and *apoBb.1* mutants injected with *apoBa* MO (K) show dark yolk (I) and complete absence of lipids in circulation (J,K) at 3 dpf .**(L)** Kaplan–Meier curves showing significant decrease in *apoBb.1* mutant viability at 60 dpf (N=3, n_{wt}=127, $n_{apoBb.1-/-}$=130). **(M)** Reduced weight of *apoBb.1* homozygous mutants at 60 dpf, as compared to wt siblings (N=3, n_{wt}=87, $n_{apoBb.1-/-}$ =37). Scale bar: (A-C) 500 µm, (D-I) 100µm, P Value: *<0.05.

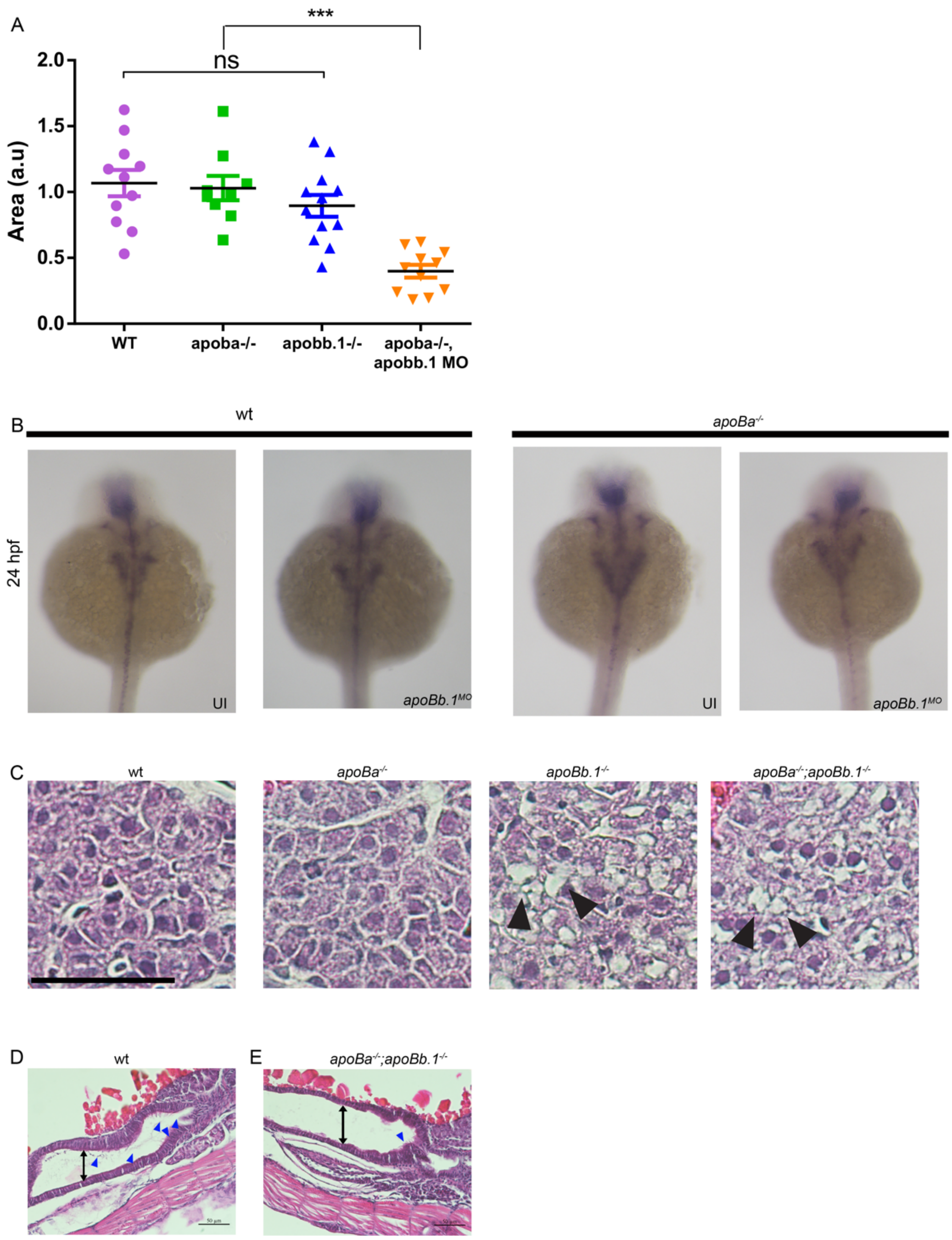

Supplemental Figure 3. (A) Liver area in wt, *apoba*$^{-/-}$, *apoBb.1*$^{-/-}$ and *apoba*$^{-/-}$*;apoBb.1*^{MO} embryos. **(B)** WISH at 24 hpf shows no differences in the expression of *foxa2* in *apoBb.1*^{MO} and *apoba*$^{-/-}$*;apoBb.1*^{MO} embryos as compared to wt and *apoba*$^{-/-}$ siblings. **(C)** H&E stained sections of 4 dpf embryos showing lipid accumulation in *apoBb.1*$^{-/-}$ and *apoba*$^{-/-}$*;apoBb.1*$^{-/-}$ embryos. Arrowheads point to lipid droplets within hepatocytes. **(D,E)** H&E staining of histological sections of the guts of wt (D) and *apoba*$^{-/-}$*;apoBb.1*$^{-/-}$ embryos (E). Blue arrowheads point to microvilli in epithelial cells. Scale bar: (C,D,E) 50µm. P Value: ***<0.001

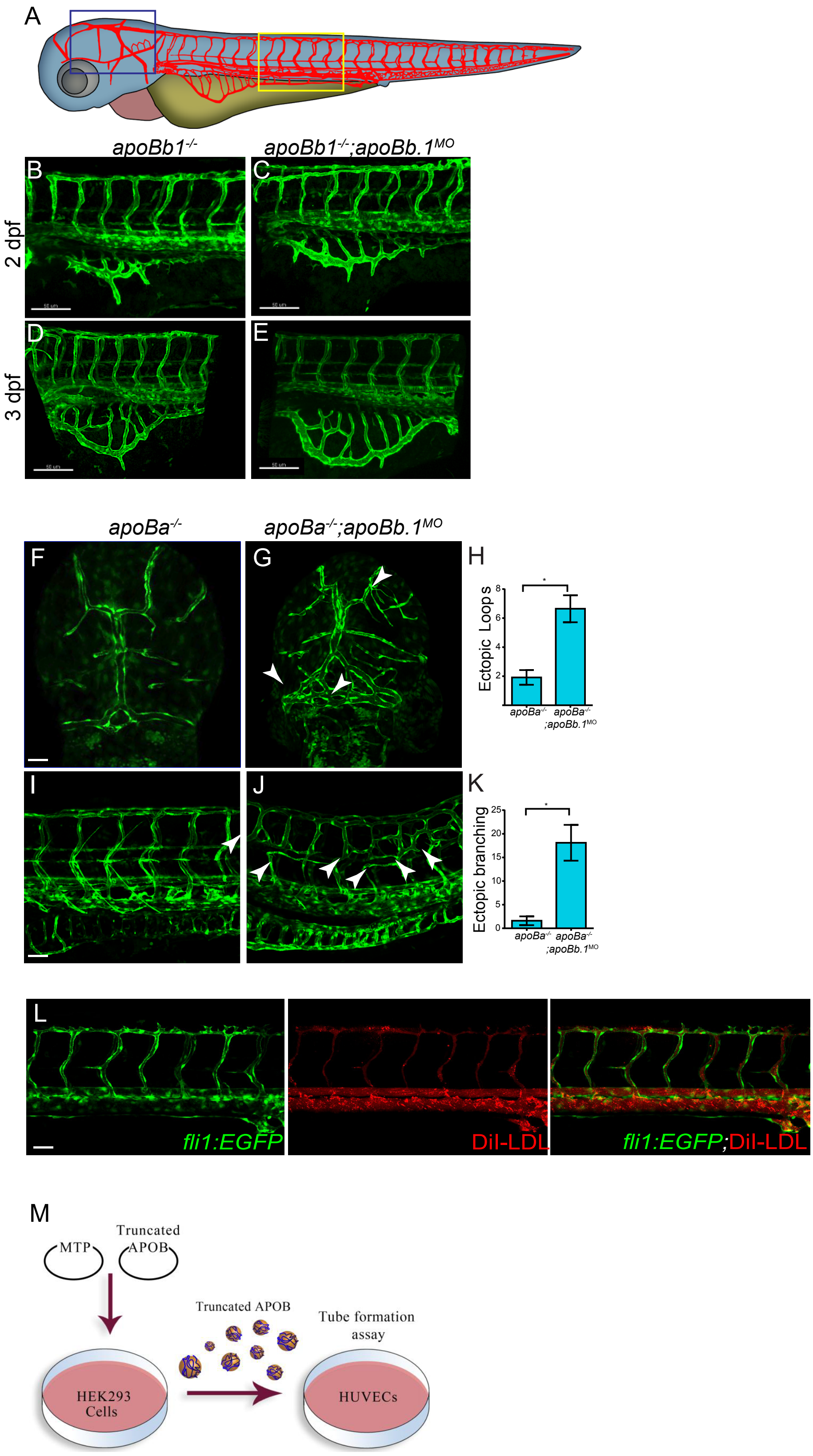

Supplemental Figure 4: (A) Schematic representation of the zebrafish embryonic vasculature, with blue square marking the cranial vasculature (shown in F,G), and yellow square depicting the trunk vasculature (I,J,L). **(B-E)** Confocal images of *apoBb.1*$^{-/-}$ at 2 (B,C) and 3 (D,E) dpf, uninjected (B,D) or injected with *apoBb.1* MO showing no defects caused by MO injection. (F,G) Dorsal view of the cranial vessels at 4 dpf showing the presence of ectopic vascular loops in the PCeV of

Tg(fli1:eGFP);*apoBa*$^{-/-}$;*apoBb.1*^{MO} (arrowheads); quantified in (H) (N=3, $n_{apoBa-/-}$=15, $n_{apoBa-/-;apoBb.1MO}$=19). **(I,J)** Confocal images of the trunk of 5 dpf *Tg(fli1:eGFP);apoBa*$^{-/-}$ (I) and *Tg(fli1:EGFP);apoBa*$^{-/-}$;*apoBb.1*^{MO} (J) exhibiting ectopic angiogenic sprouts (arrowheads) arising in the ISVs, quantified in (K) (N=3, $n_{apoBa-/-}$=14, $n_{apoBa-/-;apoBb.1MO}$=21). **(L)** Confocal images of 2 dpf *Tg(fli:eGFP)*^{y1} embryo injected intravascularly with DiI-LDL, showing proper distribution of DiI-LDL in circulation. **(M)** Schematic model describing the experimental system for production of truncated ApoB fragments.Scale bar: (A-J) 50µm, (L) 30µm, P Value: *<0.05.
